# Cell therapy as a treatment of secondary lymphedema: a systematic review and meta-analysis

**DOI:** 10.1186/s13287-021-02632-y

**Published:** 2021-11-20

**Authors:** Hector Lafuente, Ibon Jaunarena, Eukene Ansuategui, Arantza Lekuona, Ander Izeta

**Affiliations:** 1 Tissue Engineering Group, Biodonostia Health Research Institute, 20014 San Sebastián, Spain; 2 Gynecology Oncology Unit, Donostia University Hospital, 20014 San Sebastián, Spain; 3 Obstetrics and Gynaecology Group, Biodonostia Health Research Institute, 20014 San Sebastián, Spain; 4 Clinical Epidemiology Group, Biodonostia Health Research Institute, 20014 San Sebastián, Spain; 5 School of Engineering, Tecnun-University of Navarra, 20009 San Sebastián, Spain

**Keywords:** Stem cells, Lymphatic vasculature, Lymphedema, Regeneration, Regenerative medicine, Systematic review, Meta-analysis

## Abstract

**Background:**

Lymphedema, the accumulation of interstitial fluid caused by poor lymphatic drainage, is a progressive and permanent disease with no curative treatment. Several studies have evaluated cell-based therapies in secondary lymphedema, but no meta-analysis has been performed to assess their efficacy.

**Methods:**

We conducted a systematic review and meta-analysis of all available preclinical and clinical studies, with assessment of their quality and risk of bias.

**Results:**

A total of 20 articles using diverse cell types were selected for analysis, including six clinical trials and 14 pre-clinical studies in three species. The meta-analysis showed a positive effect of cell-based therapies on relevant disease outcomes (quantification of edema, density of lymphatic capillaries, evaluation of the lymphatic flow, and tissue fibrosis). No significant publication bias was observed.

**Conclusion:**

Cell-based therapies have the potential to improve secondary lymphedema. The underlying mechanisms remain unclear. Due to relevant heterogeneity between studies, further randomized controlled and blinded studies are required to substantiate the use of these novel therapies in clinical practice.

## Background

As knowledge on the diverse lymphatic vasculature roles in health and disease progresses, it increases the lymphatic vessel relevance in understanding the physiopathology of a number of diseases [1]. Lymphedema is a chronic edema, lasting more than three months, due to the accumulation of interstitial fluid caused by poor lymphatic drainage [2]. Secondary lymphedema is due to obstruction or infiltration of the lymphatic vessels by tumors, infections (recurrent lymphangitis), obesity, surgery or overload and saturation of the lower limb venous system [3]. The most frequent cause in undeveloped countries is filariasis, while in developed countries, it is iatrogenic due to radiotherapy or surgery related to the management of malignant neoplasms (breast cancer, malignant melanoma, gyneco-urological cancer) [4]. Approximately, 30% of women with breast cancer and 20% of melanoma patients who have axillary and inguinal lymph nodes removed, develop lymphedema [5, 6].

The accumulation of lymph in the interstitial tissue leads to remodeling of the skin and subcutaneous tissue and the accumulation of fibroadipose tissue [7]. The chronic form of lymphedema is characterized by swelling, fibrosis, accumulation of adipose tissue and infiltration of immune cells. Clinically, it can be classified into four stages: in stage 0, the condition is considered subclinical; swelling is not present. In stage I, edema is mild; fluid accumulates throughout the day but resolves overnight. In stage II, lymphedema is always present, but varies in severity. Stage III disease is characterized by persistent moderate-to-severe edema in the affected limb [8].

Lymphedema is a progressive and permanent disease for which there is no curative treatment. The standard treatment is physiotherapy (lymphatic drainage and compression bandaging) [9], although other treatments used include pharmacotherapy and surgery. More recently, reconstructive microsurgery (lympho-venous anastomosis, lymphatic vessel transplantation and autologous lymph node transplantation) has been proposed as an alternative [10–12].

Other potential therapies are still in development, e.g., the therapeutic potential of different growth factors, which would facilitate the regrowth of damaged, dysfunctional or obliterated lymphatics, has been investigated [13, 14]. Among them, the role of vascular endothelial growth factor VEGF-C as a stimulant of lymphangiogenesis and mediator of lymphatic endothelial cell growth and viability has been studied [15], as well as fibroblast growth factor-2 and hepatocyte growth factor [16]. Also, the use of gene therapy via adenovirus, plasmids or even direct application of recombinant VEGF-C has been described to reduce edema in different preclinical models [17–19]. However, there are currently many unresolved questions, such as the lifespan of recombinant proteins, the time-limited action of gene therapy, as well as the side effects of growth factors on the blood vasculature and on the development of new tumors [18, 20].

In the last decade, cell therapy with differentiated or progenitor cells has emerged as a new research target in the therapy of secondary lymphedema [21, 22]. Although the cellular pathways through which stem cell therapy could help lymphedema patients are unclear, in vitro studies indicate that stem cells may differentiate into lymphatic endothelial-like cells under in vitro culture conditions and can improve interstitial fluid drainage when injected in vivo [13]. Stem cells have a wide range of therapeutic effects in terms of anti-inflammation, anti-fibrosis, anti-oxidative stress, as well as promoting the regeneration of different tissues. These properties could promote the regeneration of lymphatic vessels, rebuild lymphatic circulation and successfully treat lymphedema. Currently, several clinical and preclinical studies have evaluated the therapeutic potential of using lymphatic endothelial progenitor cells (LEPCs), embryonic stem cells (ESCs), induced pluripotent stem cells (iPSCs) or mesenchymal stromal cells (MSCs) in the regeneration of lymphatic vessels. These results suggest that stem cell therapy is feasible and may promote recovery in patients with secondary lymphedema. However, stem cell transplantation has not been fully evaluated for the treatment of secondary lymphedema in clinical settings. In the present study, a meta-analysis of the available data was performed to evaluate the safety and efficacy of stem cell therapy for the treatment of secondary lymphedema.

## Methods

We conducted a systematic review according to the Cochrane method [23] and SYRCLE guideline [24], and the results are reported in accordance with PRISMA guidelines [25]. The protocol for this review was registered on the International prospective register of systematic reviews website (https://www.crd.york.ac.uk/prospero/) with two separate IDs (CRD42020180348 for preclinical studies and CRD42019130951 for clinical studies).

### Search strategy and literature selection

Studies of cell therapy as a treatment of secondary lymphedema were identified from Medline, Web of Science, EMBASE, and The Cochrane library with no language or time restrictions using these search terms: lymphedema, lymphoedema, lymphangiogenesis, lymphatic diseases, lymphatic vessels, lymph nodes, stem cells, stromal cells, mesenchymal stem cells, cell- and tissue-based therapy, cell transplantation, and regenerative medicine. We identified all relevant studies or trials regardless of language or publication status (published, unpublished, in press, and ongoing). Two independent searches were conducted on January 2021, one with the inclusion criteria: pre-clinical studies and all animal models, and the other one with the inclusion criteria: clinical trials and prospective controlled studies in human.

**Fig. 1:**
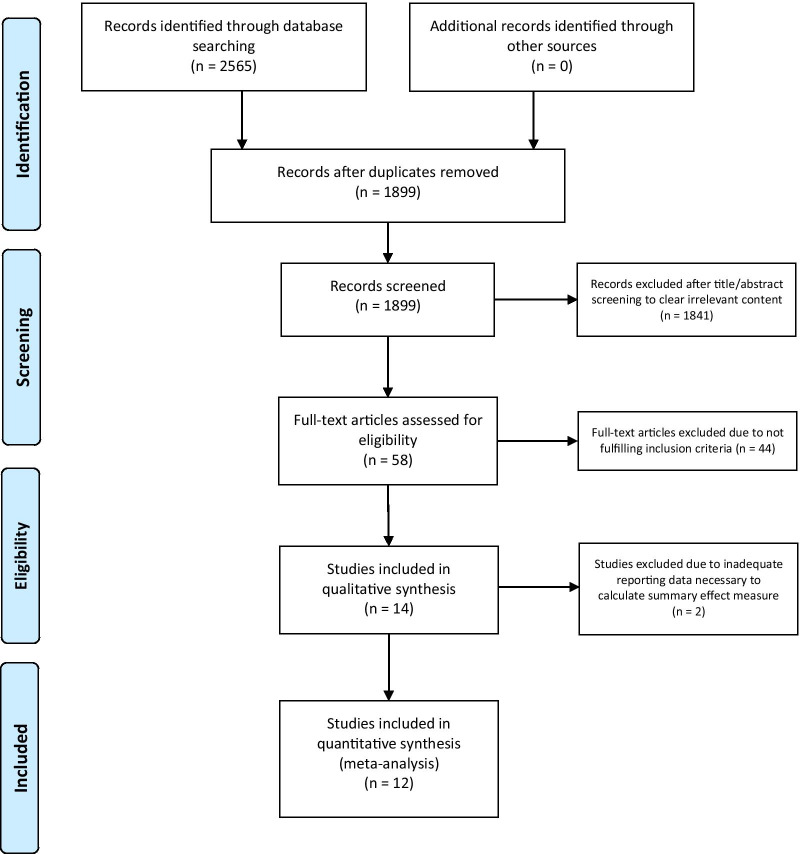
PRISMA flow diagram of search strategy and literature selection for preclinical studies

**Fig. 2:**
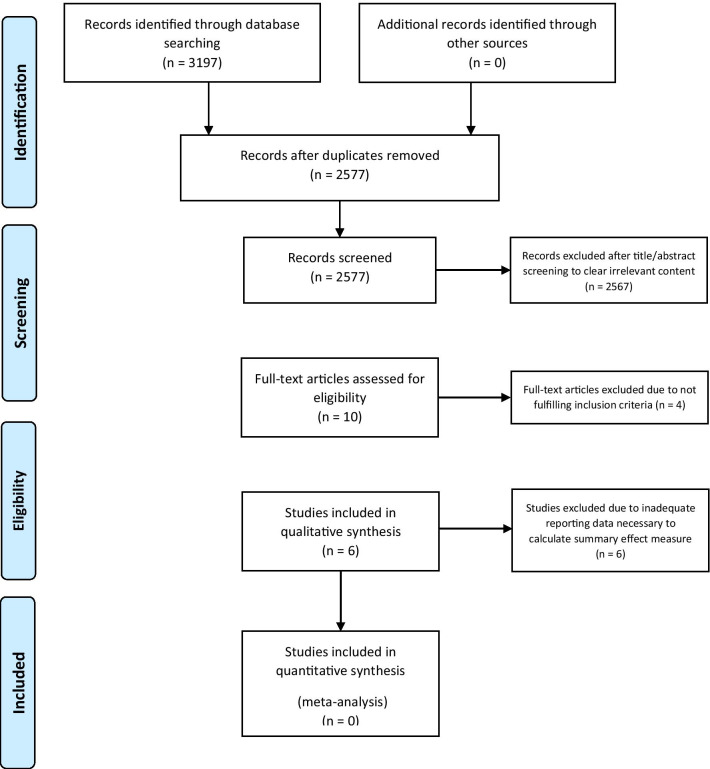
PRISMA flow diagram of search strategy and literature selection for clinical studies

After developing a search strategy for each database and collecting the citations, the search results were combined. The first selection was made using only the title and abstract of the studies. To avoid biases in the selection process, two observers independently screened articles for relevance. The criteria used for the first screening were based on the search components: (SC1) intervention (only cell therapies were included); (SC2) disease of interest (secondary lymphedema); (SC3) type of study (only pre-clinical studies, randomized controlled clinical trials and prospective controlled studies were included. The ex vivo studies, in vitro studies, or in silico studies were not included. Non-intervention studies, no control group, co-intervention studies and studies with other outcomes were not included); and (SC4) publication types (reviews and conference abstracts were not included). Only clearly irrelevant citations were removed. Citations resulting from the first screening underwent a second screening based on the predefined inclusion and exclusion criteria. Throughout the potentially relevant article selection process, the reasons for the removal of citations were documented and reported to facilitate transparency and to independently examine the accuracy of the study removal. Two independent reviewers performed all stages of the review process. Discrepancies were resolved by consensus. The flow diagram of search strategy and literature selection is shown in Fig. 1 for preclinical studies and in Fig. 2 for clinical studies.

### Assessment of study quality and risk of bias

Quality and risk of bias was assessed for clinical trials by use of Cochrane's risk of bias tool [26], and for non-randomized studies by use of NewcastleOttawa's risk of bias tool [27]. For preclinical studies, we used SYRCLE Risk of Bias tool [28]. Two authors independently assessed the risk of bias of the included studies. A third author was consulted to resolve discrepancies related to risk of bias.

Besides, to overcome the fact that there were too many items classified as “unclear” because of the poor description of details on experimental design and methods, we included three items as other bias: (a) inappropriate influence of funders, (b) mention of randomization at any level, and (c) mention of blinding at any level. For inappropriate influence of funders, “Yes” indicated non-industry source of funding, no funding, or no conflict of interest, “No” indicated the study was funded by industry- or author-mentioned conflict of interests, “unclear” indicated funding source or conflict of interest was not mentioned. For mention of randomization or blinding, “Yes” indicated reported and “No” indicated unreported.

An overall score was calculated by adding all the items scores as yes equals one, while no and not applicable equal zero. A score was given for every paper to classify them as poor, fair, or good conducted studies, where a score from 0 to 5 was considered poor, 6–9 as fair, and 10–14 as good.

### Data extraction

For clinical studies, details about the study design, cell type, primary outcome assessment, follow-up time and results were extracted.

Data on animal model characteristics (animal species, total sample size, total groups, number of animals in control group, number of animals in intervention group), lymphedema model (tail, hindlimb, etc.), cell administration characteristics (cell type, source, as well as administration route, dose, timing and anatomical site of intervention), and primary outcome measures (evaluation of the lymphatic flow, quantification of edema, density of lymphatic capillaries and tissue fibrosis) were extracted.

For included articles, all independent comparisons were identified. Replications were also collected separately. Information on primary outcome was extracted from both text and graphs, when raw data or mean/median/incidence, SD/SE were reported or recalculated. In several studies, the results were adapted to be able to be analyzed with the rest of the studies. Gsys 2.4.6. software (Hokkaido University Nuclear Reaction Data Centre) was used to obtain values from graphs. When the number of animals was reported as a range, the lowest group size was collected. When no clear data could be extracted, the report was excluded from further meta-analysis.

### Statistical analyses

Quantitative analysis was conducted using Review Manager (RevMan) version 5.3 software (The Nordic Cochrane Centre, The Cochrane Collaboration, Copenhagen, Denmark). Treatment effects were first calculated separately for each study outcome. For all analyses, a random effect, inverse variance model was used to calculate standardized mean differences (SMD) and 95% confidence intervals (CI). Because most animal experiments use fewer than ten animals per group, we used Hedge's G effect sizes (which is based on Cohen’s D but includes a correction factor for small sample size bias) for SMD analyses. The effect of heterogeneity (I^2^) was used to measure the degree of inconsistency across pooled studies due to variability rather than chance, with larger values indicative of high heterogeneity (0–25% is considered to reflect very low heterogeneity; 25–50% reflects low heterogeneity; 50–75% reflects moderate heterogeneity; > 75% reflects high heterogeneity). Considering the anticipated heterogeneity, random effects models were used to conducted meta-analysis. Mean effect size, 95% confidence intervals (95% CI), significance, weight and forest plots were analyzed by the inverse variance method and the standard mean differences. The possibility of publication bias was assessed by analyzing funnel plot asymmetry (with trim-and-fill). The trim-and-fill method provides an estimate of the number of missing studies, and also provides an estimated intervention effect ‘adjusted’ for the publication bias (based on the filled studies). Finally, to explore sources of heterogeneity, stratified meta-analysis and meta-regression were performed.

## Results

A total of 20 articles were selected for analysis. Six of these were clinical studies [29–34], including a randomized clinical trial, three nonrandomized clinical trials and two prospective controlled studies (Table 1). A case report and an observational study were excluded from the analysis. Five of them studied the effect of cell therapy on the upper limb, while the other studied lower limb edema. Mesenchymal stromal cells (MSCs) of different origins were used: three studies used bone marrow-derived MSCs (BM-MSCs) and the remaining three used adipose-derived MSCs (ADSCs). The follow‐up period ranged from 3 to 12 months.

**Table 1:** Cell‐therapy for secondary lymphedema: clinical studies

Year	References	Study type	Edema location	Cell type/dose	Follow-up/assessment	Results	Conclusions
2008	Hou et al. [29]	Prospective controlled study	Upper limb	Freshly isolated bone marrow stromal cells N/A	12 months/Circumference measurements, volume of edema, pain in arm	BM-MSCs reduce the volume and % volume of lymphedema, and reduce the amount of pain caused by edema	Autologous BM-MSCs transplantation for the treatment of breast cancer-related arm lymphedema is effective and feasible
2011	Maldonado et al. [30]	Prospective controlled study	Upper limb	Freshly isolated bone marrow stromal cells (7 – 56 × 10^7^)	3 months/Circumference measurements, chronic pain, arm mobility and sensory loss	BM-MSCs reduce the volume of lymphedema. Chronic pain and sensitivity are markedly improved	The use of localized injections of BM-MSCs appears to be helpful in the management of lymphedema secondary to radical mastectomy
2016	Toyserkani et al. [31]	Nonrandomized clinical trial	Upper limb	Freshly isolated autologous adipose-derived stromal cells (4.07 × 10^7^)	4 months/Circumference measurements, dual-energy X-ray absorptiometry scans, adverse events	ADSCs do not reduce the volume of lymphedema. Patients reported a decrease in symptoms over time. Five patients reduced their use of conservative treatment	ADSC-assisted lipotransfer is safe during the 4-month follow-up period and can alleviate symptoms of breast cancer-related lymphedema, minimizing the need for conservative treatment
2017	Toyserkani et al. [32]	Nonrandomized clinical trial	Upper limb	Freshly isolated autologous adipose-derived stromal cells (5.37 × 10^7^)	6 months/Circumference measurements, dual-energy X-ray absorptiometry scans, patient-reported outcome and safety questionnaire assessment	ADSCs do not reduce the volume of lymphedema. Patients reported a decrease insymptoms over time. Five patients reduced their use of conservative treatment	ADSC-assisted lipotransfer is safe during the 6-month follow-up period and can alleviate symptoms of breast cancer-related lymphedema, minimizing the need for conservative treatment
2018	Ismail et al. [33]	Randomized controlled trial	Lower limb	Freshly isolated bone marrow stromal cells N/A	6 months/Circumferential measurements, heaviness and pain improvement, Immunohistochemical staining (lymphangiogenesis), recurrence of lymphedema	BM-MSCs reduce edema circumference as well as pain relief and improvement in walking ability. Increase in the number of lymphatic capillaries	BM-MSCs treatment can achieve improvement of symptoms in patients with chronic lymphedema
2019	Toyserkani et al. [34]	Nonrandomized clinical trial	Upper limb	Freshly isolated autologous adipose-derived stromal cells (5.41 × 10^7^)	12 months/Circumference measurements, dual-energy X-ray absorptiometry scans, Patient-reported outcome and safety questionnaire assessment, lymphoscintigraphy changes	ADSCs do not reduce the volume of lymphedema. Patients reported a decrease in symptoms over time. Five patients reduced their use of conservative treatment	ADSC-assisted lipotransfer is safe during the 12-month follow-up period and can alleviate symptoms of breast cancer-related lymphedema, minimizing the need for conservative treatment

Fourteen animal studies [35–48] were included in the analysis (Table 2). These studies included three different animal models (mouse, rat and rabbit). In murine models, tail, hind limb, back skin flap, or lymph node transplantation was used. In rabbit models, hind limb was used. The cell types used included stem or progenitor cells (BM-MSCs, ADSCs, muscle‐derived stem cells and multipotent progenitor cells) and differentiated cells (lymphatic endothelial cells and T_reg_ cells), and the number of cells used ranged from 10^4^ to 10^7^. The follow‐up period ranged from 14 days to 6 months.

**Table 2:** Cell therapy for secondary lymphedema: non-clinical studies

Year	References	Animal model	Groups	Cell type/number	Implantation methods	Follow-up/Assessment	Results	Conclusions
2009	Conrad et al. [35]	Mouse tail	2 groups (*n* = N/A for each group): Control, MSC	Allogeneic up to 3 passages BM-MSC (p53 ^−/−^)/1 × 10^7^	Subcutaneous	56 days/Circumference measurements, lymphatic drainage, neolymphangiogenesis (immunohistochemical staining)	(1) In stem cell-treated animals, a marked reduction in the edema was observed (2) Restoration of lymphatic drainage	The administration of BM-MSCs in vivo may contribute to the reduction in lymphatic edema
2011	Hwang et al. [36]	Mouse hindlimb	5 groups (*n* = 5): Sham, control, hydrogel alone, hADSC, hADSC + hydrogel	PKH-26-labeled hADSC/VEGF-C hydrogel/N/A	Subcutaneous	28 days/Circumference mesurements, lymphatic vessels (immunohistochemical staining)	(1) Significantly decreased dermal edema depth (2) Significantly greater lymphatic vessel regeneration	Co-administration of hADSCs and VEGF-C hydrogel has a substantial positive effect on lymphangiogenesis
2011	Zhou et al. [37]	Rabbit Hindlimb + IR	4 groups (*n* = N/A): Control, VEGF-C, BM-MSC, BM-MSC + VEGF-C	Allogeneic 3 passages BM-MSC + VEGF-C/1 × 10^7^	Intramuscular	6 months/Limb volume changes, Immunohistochemical staining of lymphatic vessels, western blot analysis for VEGF-C	(1) Reduce limb volume at 6 months (2) Significant greater lymphatic vessel at 28 days	BM-MSC transplantation and VEGF-C administration could enhance the therapeutic effect of each other
2012	Shimuzu et al. [38]	Mouse tail	5 groups (*n* = 12): Sham, PBS, VEGF-C, BM-MNC, ADSC	Freshly isolated ADSCs/2 × 10^6^	Subcutaneous	28 days/Tail diameter, lymphatic vessels diameter (H-E), lymphatic vessels (immunohistochemical staining), bone marrow-derived CD11b + macrophage kinetics assay	(1) Lymphedema was improved significantly by local injection of ADSCs (2) High lymphatic capillary density (3) Enhance recruitment of bone marrow-derived M2 macrophages, which serve as lymphatic endothelial progenitor cells	Implantation of autologous ADSCs could be a useful treatment option for patients with severe lymphedema via enhanced lymphangiogenesis
2013	Park et al. [39]	Mouse Hindlimb + IR	4 groups (*n* = 8): Control, Surgery, Surgery + IR, Cell therapy	Allogeneic muscle-derived stem cell + hLEC /1 × 10^7^	N/A	56 days/Water displacement volumetric analysis, lymphoscintigraphy, lymphatic vessels (immunohistochemical staining),	(1) Attenuation of hindlimb volume (2) High lymphatic vessel density (3) Restore of the lymphatic flow	Stem cell lymphangiogenesis seems to be a promising approach
2014	Kawai et al. [40]	Nude rat tail	4 groups: hLEC (*n* = 18), hDMEC (*n* = 8), Control (*n* = 19), sham (*n* = 5)	Human dermal microvascular endothelial cells (hDMEC) and human lymphatic endothelial cells (hLEC)/5 × 10^6^	Wound/on postoperative days 1, 4, 7, 11 and 14	36 days/Circumference mesurements, indocyanine green fluorescence lymphography, thickness of epidermis (H-E), lymphatic vessels (immunohistochemical staining)	(1) In hLEC-treated animals, the circumference, lymphatic flow, and thickness of the skin became thinner (2) High lymphatic vessel density (3) hLECs are incorporated into the new vessels	Cell transplantation therapy using human LECs improved secondary lymphedema
2015	Ackermann et al. [41]	Mouse tail	3 groups (*n* = 10): Control, PRP, ADSC	Allogeneic 3 passages ADSC vs platelet-rich plasma (PRP)/N/A	N/A	14 days/Wound healing analysis, tail diameter, real-time laser Doppler imaging for perfusion, lymphatic vessels (immunohistochemical staining)	(1) PRP and ADSC show a significantly increased epithelialization (2) High lymphatic vessel density in PRP group (3) Significant enhance perfusion of wounds treated by PRP and ADSC	PRP induces higher lymphangiogenesis than ADSCs
2015	Yoshida et al. [42]	Mouse Hindlimb + IR	5 groups (*n* = 20): Sham, control, ADSC 10^4^, ADSC 10^5^, ADSC 10^6^	Allogeneic up to 5 passages ADSC/1 × 10^4^, 1 × 10^5^, 1 × 10^6^	N/A	16 days/Circumferential measurement, lymphatic flow assessment, quantification of lymphatic vessels (immunohistochemical staining and EGFP)	(1) The numbers of lymphatic vessels were significantly increased (2) ADSCs are not detected in lymphangiogenesis	ADSCs can restore the lymphatic vascular network in secondary lymphedema with increased collecting vessels
2016	Gousopoulos et al. [43]	Transgenic mice tail	2 groups (*n* = 5) Control, T_reg_	Regulatory T Cells (T_reg_)/0.8–0.9 × 10^6^	Intravenous	14 or 42 days/Tail volume, lymphatic vessels (immunohistochemical staining), RT-PCR, flow cytometry	(1) Reverse all of the major hallmarks of lymphedema, including edema, inflammation, and fibrosis (2) Promote lymphatic drainage function	T_reg_ application constitutes a potential new curative treatment modality for lymphedema
2017	Hayasida et al. [44]	Mouse Hindlimb + IR	4 groups (*n* = 5): Control, VLNT, ADSC, ADSC + VLNT	Allogeneic 1–3 passages ADSC and vascularized lymph node transfers/1 × 10^4^	Subcutaneous	14 days/Volumetric analysis of edema, near-infrared video camera system for lymphatic flow assessment, B16 mouse melanoma cells for lymphatic vessel and lymph node function, lymphatic vessels (immunohistochemical staining)	(1) ADSC + VLNT reduce the edema at 14 days (2) Increase the number of lymphatic vessels (3) Accelerate the lymphatic drainage to the venous systems	Combined ADSC and vascularized lymph node transfer treatment in secondary lymphedema may effectively decrease edema volume and restore lymphatic function
2018	Beerens et al. [45]	Nude mouse Skin flap model/Nude mouse Lymph node transplantation model	(1) Skin flaps groups (PBS *n* = 10/mMAPCs *n* = 6/hMAPCs *n* = 6) (2) Lymph node transplantation groups (PBS *n* = 10/hMAPCs 1n = 10/hMAPCs2 *n* = 6)	Allogeneic MAPCs/0.5 × 10^6^ in lymph node transplantation model; 1 × 10^6^ in skin flap model	Subcutaneous	16 weeks/lymphography, lymphatic vessels (immunohistochemical staining)	(1) Restored lymph drainage across skin flaps (2) Reconnected transplanted lymph nodes to the host lymphatic vessel	MAPC transplantation represents a promising remedy for lymphatic system restoration at different anatomical levels and hence an appealing treatment for lymphedema
2020	Bucan et al. [46]	Mouse Hindlimb + IR	3 groups (*n* = 15): Control, SVF, ADSC	Freshly isolated ADSCs vs stromal vascular fraction/1 × 10^6^	Subcutaneous	8 weeks/CT and SPECT lymphoscintigraphy for volumetric measures, lymph vessel morphometry	(1) Treatment with ADSC did not reduce the edema at 8 weeks (2) lymph vessel lumen decreased when treated with ADSC	ADSC did not improve secondary lymphedema in this animal model
2020	Dai et al. [47]	Mouse Hindlimb	4 groups (*n* = 5): Control, ADSC unsorted, ADSC Pod+, ADSC Pod−	Freshly isolated ADSCs (Pod+, Pod−) / 2 × 10^6^	Subcutaneous	10 weeks/Limb volume change, lymphatic vessels (immunohistochemical staining)	(1) More attenuation of hindlimb volume in Pod + cells (2) High lymphatic vessel density	The podoplanin-positive cells possessed lymphatic paracrine and differentiation abilities and may represent LEPCs
2020	Ogino et al. [48]	Mouse Hindlimb + IR	3 groups (*n* = 6): Control without IR, Control with IR, ADSC	Allogeneic 2–4 passages ADSCs/7.5 × 10^5^	Subcutaneous	14 days/lymphatic vessels (immunohistochemical staining), picrosirius red staining for fibrosis	(1) ADSC transplantation accelerated LEC proliferation and increased lymphatic vessel numbers (2) ADSC mitigated fibrosis	ADSC transplantation contributes to lymphedema reduction by promoting LEC proliferation, improving fibrosis and increasing the number of lymphatic vessels

### Assessment of study quality and risk of bias

The study design, including details of the method of randomization of subjects to treatment groups, criteria for eligibility in the study, blinding, method of assessing the outcome, and handling of protocol deviations are important features defining study quality. Due to the high risk of bias (data not shown) and the fact that only one of the human studies included was a properly blinded randomized controlled trial, a meta-analysis was not performed for clinical studies.

**Fig. 3:**
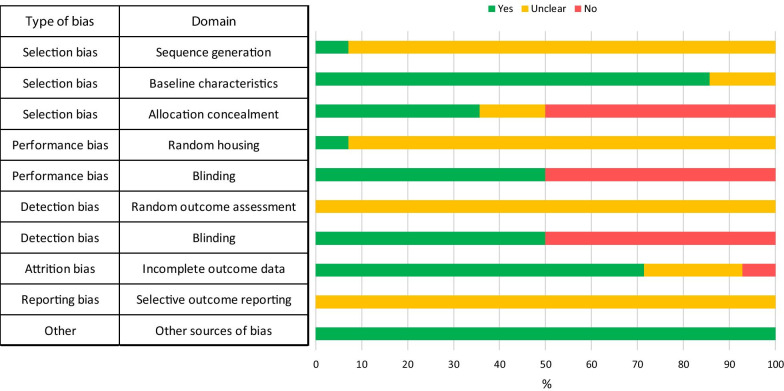
Assessment of bias in 14 animal studies using the SYRCLE risk of bias tool

None of the pre-clinical studies had published protocols nor were registered with CAMARADES (University of Edinburgh, Scotland). Therefore, the selective outcome reporting item on the SYRCLE tool was scored as “unclear.” There was insufficient information reported for many of the remaining nine questions which were scored as “unclear.” Several studies reported any randomization, although details were not given. 50% reported any blinding, either of investigators, animal handlers or outcome assessors. Overall, all studies had significant risks of bias according to the SYRCLE tool (Fig. 3), but these were not sufficiently remarkable as to be excluded from any analyses. Only two studies (Conrad et al. and Zhou et al.) did not report sample size for control and intervention groups, and thus those studies were not included in the meta-analysis.

### Meta-analysis and effect evaluation

Meta-analysis was performed for outcomes that had data in at least three studies. The outcomes analyzed were: quantification of edema, density of lymphatic capillaries, evaluation of the lymphatic flow, and tissue fibrosis.

- Quantification of edema

**Fig. 4:**
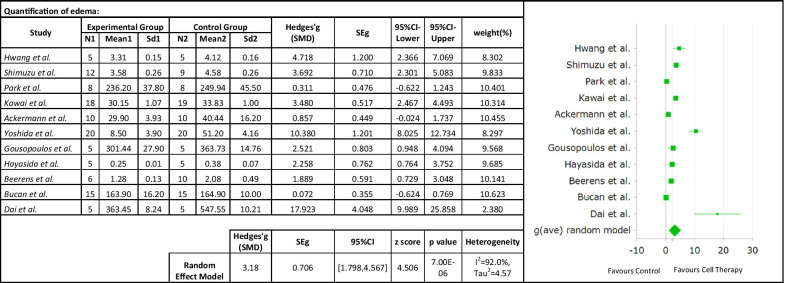
Forest plot of the effects of cell therapy on the edema reduction

**Fig. 5:**
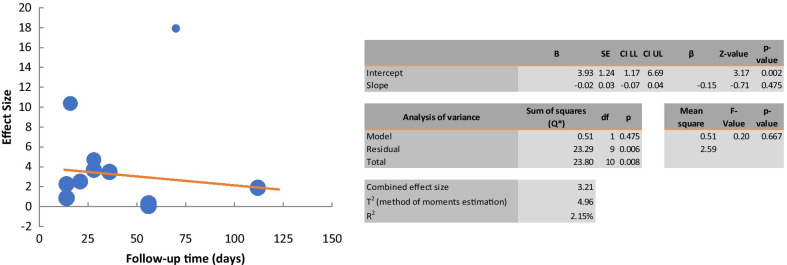
Meta-regression analysis of follow-up time on effect of the cell therapy on the edema reduction

Eleven studies were included to investigate the effect of cell therapy treatment on edema reduction in secondary lymphedema. The pooled estimate showed a significant decrease in edema (SMD 3.18; 95% CI 1.798, 4.567 (*p* < 0.001); however, between-study heterogeneity was very high (*I*^2^ = 92%; Fig. 4). Subgroup analysis as a function of the animal model used did not reduce heterogeneity. Subgrouping as a function of cell type indicated a similar reduction in edema with stem or progenitor cell treatment than differentiated cell treatment, with no evidence of heterogeneity in this subgroup (Table 3). Random effect meta-regression analysis was applied to estimate functional relationship of effect size on follow-up time. The regression coefficient was -0.02, and it was statistically insignificant (*p* > 0.05). These results indicated that the effect of follow-up time on the effect size was insignificant. Consistently, a linear relationship was not found (Fig. 5).

- Density of lymphatic capillaries

**Table 3:** Subgroup analyses of the effects of cell therapy on secondary lymphedema

Subgroup	Experiments (N)	Hedges' G (SMD)	SEg	95%CI-Lower	95%CI-Upper	z score	*p* value	Heterogeneity %
(1) Edema								
All studies	11	3.183	0.706	1.798	4.567	4.506	0.000	92.250
Animal model								
Tail model	5	3.330	0.963	1.442	5.217	3.458	0.001	88.307
Hindlimb model	5	3.329	1.315	0.751	5.907	2.531	0.011	95.132
Cell type								
Stem or progenitor cells (BM-MSC, ADSC, MAPC)	9	3.282	0.831	1.652	4.911	3.948	0.000	92.932
Differentiated cells (LEC, T_reg_)	2	3.197	0.438	2.339	4.055	7.305	0.000	0.989
(2) Lymphatic vessels								
All studies	10	6.348	1.139	4.115	8.581	5.571	0.000	92.650
Animal model								
Tail model	4	6.661	2.157	2.434	10.889	3.089	0.002	95.592
Hindlimb model	5	5.736	1.243	3.299	8.173	4.613	0.000	84.502

**Fig. 6:**
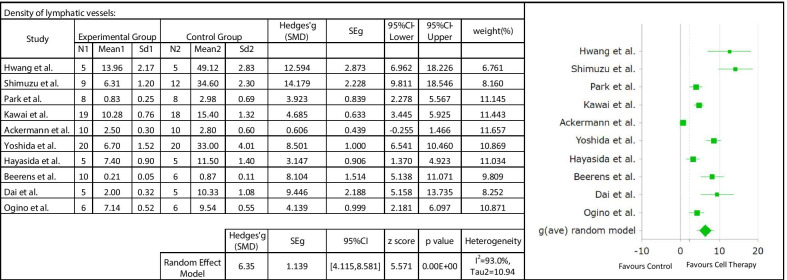
Forest plot of the effects of cell therapy on the lymphatic regeneration

**Fig. 7:**
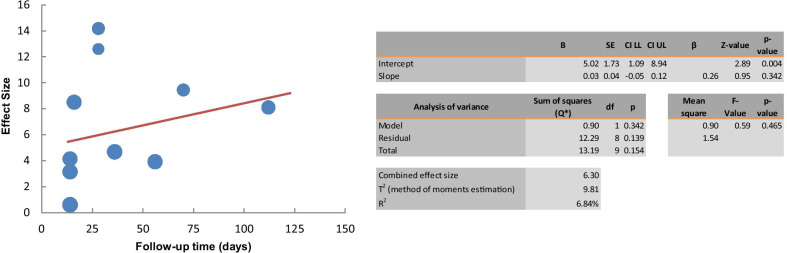
Meta-regression analysis of follow-up time on effect of the cell therapy on the lymphatic regeneration

Ten studies were included to investigate the effect of the cell therapy treatment on the lymphatic regeneration in secondary lymphedema. The overall pooled analysis showed a significant increase in lymphatic vessel density in experimental group versus control group (SMD 6.35; 95% CI 4.115, 8.581; *p* = 0.00). However, the test for heterogeneity was significant (*I*^2^ = 93%; Fig. 6). Subgroup analysis as a function of animal model did not show differences between groups and did not reduce heterogeneity. Analysis as a function of cell type could not be carried out due to the small number of studies (Table 3). Random effect meta-regression analysis was applied and a regression coefficient of 0.03 was found, which was statistically insignificant (*p* > 0.05). These results indicated that follow-up time does not explain the heterogeneity found between the studies (Fig. 7).

- Evaluation of the lymphatic flow

**Fig. 8:**
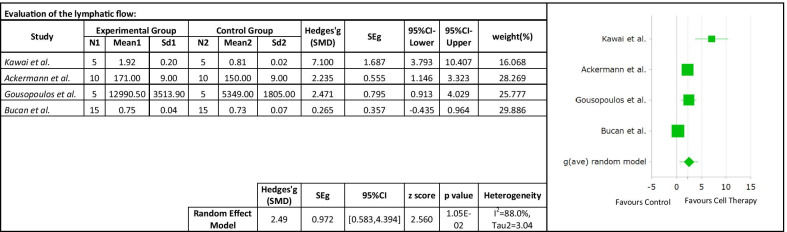
Forest plot of the effects of cell therapy on the lymphatic perfusion restoration

**Fig. 9:**
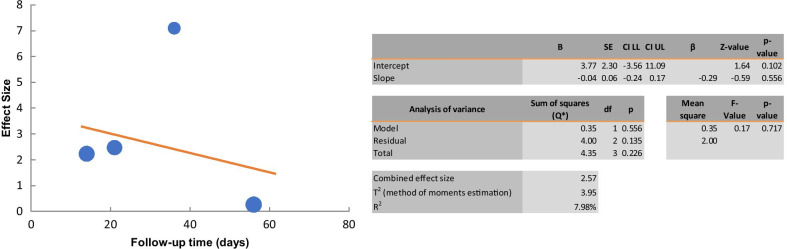
Meta-regression analysis of follow-up time on effect of the cell therapy on lymphatic perfusion restoration

Four studies were included to investigate the effect of the cell therapy treatment on the lymphatic perfusion restoration in secondary lymphedema. The pooled estimate suggested a significant improvement of lymphatic perfusion (SMD 2.49; 95% CI 0.583, 4.394 (*p* = 0.01); *I*^2^ = 88%, Fig. 8). Due to the limited availability of data, it was not possible to conduct subgroup analyses. Using random effects meta-regression analysis, the regression coefficient was -0.04, which was not statistically significant (*p* > 0.05). The results indicated that follow-up time does not explain the heterogeneity between studies (Fig. 9).

- Tissue fibrosis

**Fig. 10:**
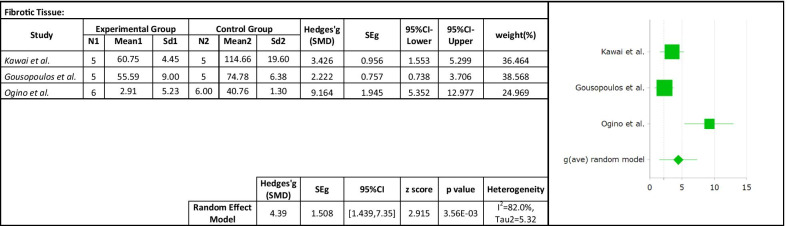
Forest plot of the effects of cell therapy on the fibrosis reduction

**Fig. 11:**
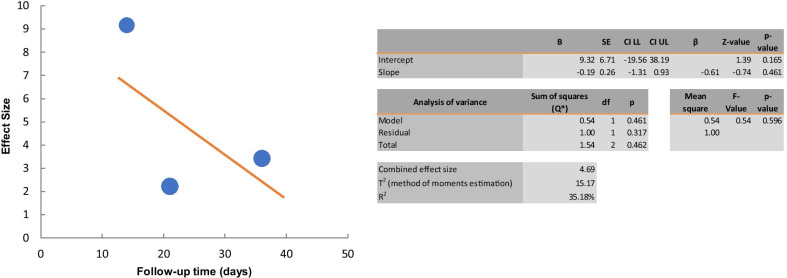
Meta-regression analysis of follow-up time on effect of the cell therapy on the fibrosis reduction

Only three studies were included to investigate the effect of cell therapy treatments on the fibrosis reduction in secondary lymphedema. The analysis of the effect size showed a significant reduction in the fibrosis (SMD 4.39; 95% CI 1.439, 7.352 (*p* < 0.01); *I*^2^ = 82%, Fig. 10). Subgroup analysis was not carried out due to the small number of studies included. The regression coefficient was found to be -0.19 and statistically insignificant (*p* > 0.05) using random effect meta-regression analysis. The study's heterogeneity was not explained by the follow-up period, according to the findings (Fig. 11).

### Publication Bias

**Fig. 12:**
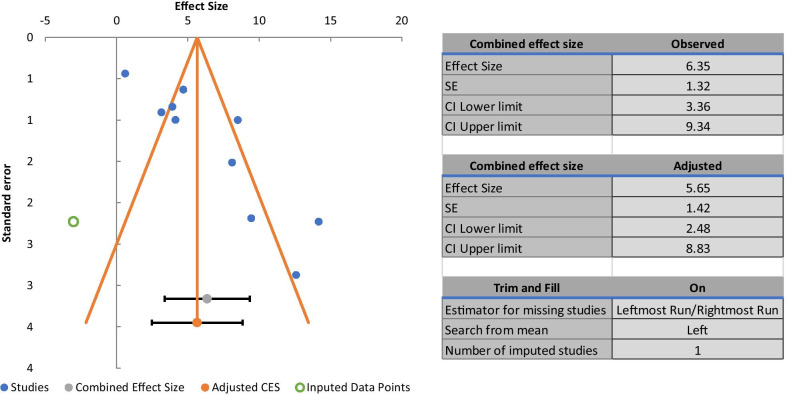
Funnel plot (with trim-and-fill) analysis of the cell therapy on the lymphatic regeneration

The publication bias evaluation (Funnel plots) for the meta-analysis of lymphatic regeneration (ten studies) is shown in Fig. 12. After adjusting for missing studies, we found that the point estimate of the overall effect size continued to show a positive effect in favor of cell therapy (SMD 5.65 [CI 95% 2.48–8.83]). No significant publication bias was observed for edema reduction, lymphatic perfusion restoration and fibrosis reduction. This confirms that if there were a publication bias, the effect of cell therapy on secondary lymphedema would not be modified.

## Discussion

In the present study, we performed a systematic review and meta-analysis to evaluate the safety and efficacy of stem cell therapy for the treatment of secondary lymphedema, both in preclinical and clinical studies. We found that cell therapy proved to generate a robust beneficial effect in animal models of secondary lymphedema. Although several in vitro and in vivo studies have reported beneficial effects of cell therapy against secondary lymphedema [21, 49, 50], a formal meta-analysis that assesses the regenerative activity of cell therapy in animal models of secondary lymphedema had not been performed.

Animal studies are critical for understanding disease processes and assessing the safety and effectiveness of treatments. Animal trials, however, are inherently heterogeneous, even more than clinical trials. Understanding sources of heterogeneity and their influence on effect size is critical to successfully translating preclinical findings to human diseases [51].

No animal model mimics perfectly the pathophysiology of human lymphedema [52], mainly because animals present higher regenerative capacity and it is difficult to classify the severity of edema [49]. There are also significant differences between models [52]. Although the tail model yields more consistent results than the hindlimb model, lymphedema resolves naturally over time, thus confounding results of additional interventions [53]. Of note, the current lack of standardization in study design and outcome measures make it hard to compare preclinical results. Despite the experimental heterogeneity of available studies, insight from animal models has shed light on the molecular mechanisms underlying lymphedema, e.g., lymphangiogenesis [54], fibrosis [55] and inflammation [56, 57].

Regarding the human studies, only six studies were identified and included for the analysis, and since only one of them is a randomized clinical trial, it was not possible to perform the meta-analysis. Furthermore, the difference in the follow-up period between the studies did not allow us to confirm the observed effect of cell therapy on secondary lymphedema. However, it should be noted that the current human studies showed promising results of BM-MSCs [29, 30, 33] and ADSCs [31, 32, 34] in terms of reduction in edema, relief of symptoms, and an improved quality-of-life. Although no adverse effects related to cancer have been observed, the potential risk of cancer recurrence of using stem cells in the treatment of secondary lymphedema should be studied. A recently published Phase I study has found no evidence of breast cancer recurrence at 4-year follow up [58]. However, to further substantiate this relevant safety concern, a greater number of patients must be followed up longer-term in randomized clinical studies to formally rule out any contribution of stem cell transplants to cancer recurrence.

In the preclinical studies included in the review, different cell types have been tested for secondary lymphedema. In all cases, stem/progenitor cells have shown promise in halting lymphedema progression, sometimes even reverse the pathological process. However, the underlying mechanisms are not clear. It is speculated that stem cells may differentiate into lymphatic endothelial progenitor cells that in turn generate new lymphatics, or secrete cytokines to induce lymphangiogenesis [59]. Several studies have combined cell therapy with growth factors, such as VEGF‐C [36] and PRP [41] which are thought to costimulate lymphangiogenesis. Co-transplantation with lymphatic endothelial cells (LECs) [40] may guide differentiation of stem cells to LEPCs. Combination of cell therapy with lymph node transfer [44] improved both lymphangiogenesis and lymphatic flow. Of course, immune modulation could be another cell-based approach to tackle this disease. Gousopoulos et al. have shown that treatment with T_reg_ cells reversed major hallmarks of lymphedema, such as edema, inflammation, and fibrosis [43]. Cell-based therapies seem thus to improve lymphedema's outcomes (edema reduction, lymphatic regeneration, lymphatic perfusion restoration, and fibrosis reduction), and the effect is seen across multiple species (mouse, rat, and rabbit), so that translation of these novel therapies to humans seems to be warranted.

The main limitations of this study are (i) the significant methodological differences between studies, especially the animal model used, the number of infused cells and timing of follow-up; (ii) small sample sizes and small study dataset for the meta-analysis, with most studies having no pre-published protocols or sample size estimations; (iii) the included studies had moderate or unknown bias risks, mainly due to poor reporting detail, and (iv) lack of operator blindness and randomization. These limitations emphasize the importance of applying more rigor to reporting standards and publishing in vivo experimental protocols [60].

## Conclusions

Cell-based therapies have the potential to improve secondary lymphedema through their effects on the edema, lymphangiogenesis and fibrosis. The underlying mechanisms remain unclear. Due to relevant heterogeneity between studies, further randomized controlled and blinded studies are required to substantiate the use of these novel therapies in clinical practice.

## Data Availability

The datasets used and/or analyzed during the current study are available from the corresponding author on reasonable request.

## Abbreviations

ADSCs: Adipose-derived stem cells
BM-MSCs: Bone marrow-derived mesenchymal stem cells
ESCs: Embryonic stem cells
iPSCs: Induced pluripotent stem cells
I^2^: Effect of heterogeneity
LECs: Lymphatic endothelial cells
LEPCs: Lymphatic endothelial progenitor cells
MSCs: Mesenchymal stromal cells
SMD: Standardized mean differences
95% CI: 95% Confidence intervals

## References

[1] Oliver G, Kipnis J, Randolph GJ, Harvey NL (2020). The lymphatic vasculature in the 21^st^ century: novel functional roles in homeostasis and disease. Cell.

[2] Manrique OJ, Bustos SS, Ciudad P, Adabi K, Chen WF, Forte AJ (2020). Overview of lymphedema for physicians and other clinicians: a review of fundamental concepts. Mayo Clinic Proc..

[3] Brix B, Sery O, Onorato A, Ure C, Roessler A, Goswami N (2021). Biology of lymphedema. Biology.

[4] Cormier JN, Askew RL, Mungovan KS, Xing Y, Ross MI, Armer JM (2010). Lymphedema beyond breast cancer: a systematic review and meta-analysis of cancer-related secondary lymphedema. Cancer.

[5] McLaughlin SA, Wright MJ, Morris KT, Giron GL, Sampson MR, Brockway JP (2008). Prevalence of lymphedema in women with breast cancer 5 years after sentinel lymph node biopsy or axillary dissection: objective measurements. J Clin Oncol.

[6] Williams AF, Franks PJ, Moffatt CJ (2005). Lymphoedema: estimating the size of the problem. Palliat Med.

[7] Stritt S, Koltowska K, Makinen T (2021). Homeostatic maintenance of the lymphatic vasculature. Trends Mol Med..

[8] Morgan CL, Lee BB (2008). Classification and staging of lymphedema. Lymphedema: diagnosis and treatment.

[9] Brandao ML, Soares H, Andrade MDA, Faria A, Pires RS (2020). Efficacy of complex decongestive therapy for lymphedema of the lower limbs: a systematic review. J Vasc Bras.

[10] Tang JB, Landin L, Cavadas PC, Thione A, Chen J, Pons G (2020). Unique techniques or approaches in microvascular and microlymphatic surgery. Clin Plast Surg.

[11] Chen K, Sinelnikov MY, Shchedrina MA, Mu L, Lu P (2021). Surgical management of postmastectomy lymphedema and review of the literature. Ann Plastic Surg.

[12] Gasteratos K, Morsi-Yeroyannis A, Vlachopoulos NC, Spyropoulou GA, Del Corral G, Chaiyasate K (2021). Microsurgical techniques in the treatment of breast cancer-related lymphedema: a systematic review of efficacy and patient outcomes. Breast Cancer (Tokyo, Japan).

[13] Dayan JH, Ly CL, Kataru RP, Mehrara BJ (2018). Lymphedema: pathogenesis and novel therapies. Annu Rev Med.

[14] Forte AJ, Boczar D, Huayllani MT, McLaughlin SA, Bagaria S (2019). Topical approach to delivering targeted therapies in lymphedema treatment: a systematic review. Cureus.

[15] Cheung L, Han J, Beilhack A, Joshi S, Wilburn P, Dua A (2006). An experimental model for the study of lymphedema and its response to therapeutic lymphangiogenesis. BioDrugs.

[16] Saito Y, Nakagami H, Morishita R, Takami Y, Kikuchi Y, Hayashi H (2006). Transfection of human hepatocyte growth factor gene ameliorates secondary lymphedema via promotion of lymphangiogenesis. Circulation.

[17] Enholm B, Karpanen T, Jeltsch M, Kubo H, Stenback F, Prevo R (2001). Adenoviral expression of vascular endothelial growth factor-C induces lymphangiogenesis in the skin. Circ Res.

[18] Saaristo A, Veikkola T, Tammela T, Enholm B, Karkkainen MJ, Pajusola K (2002). Lymphangiogenic gene therapy with minimal blood vascular side effects. J Exp Med.

[19] Szuba A, Skobe M, Karkkainen MJ, Shin WS, Beynet DP, Rockson NB (2002). Therapeutic lymphangiogenesis with human recombinant VEGF-C. FASEB J.

[20] Goldman J, Le TX, Skobe M, Swartz MA (2005). Overexpression of VEGF-C causes transient lymphatic hyperplasia but not increased lymphangiogenesis in regenerating skin. Circ Res.

[21] Chen K, Sinelnikov MY, Reshetov IV, Timashev P, Gu Y, Mu L (2021). Therapeutic potential of mesenchymal stem cells for postmastectomy lymphedema: a literature review. Clin Transl Sci.

[22] Hu LR, Pan J (2020). Adipose-derived stem cell therapy shows promising results for secondary lymphedema. World J Stem Cells.

[23] Higgins JPT, Thomas J, Chandler J, Cumpston M, Li T, Page MJ, Welch VA. Cochrane handbook for systematic reviews of interventions version 6.2 (updated February 2021). Cochrane; 2021. Available from www.training.cochrane.org/handbook.

[24] Hooijmans CR, Wever KE, de Vries RBM. SYRCLEs starting guide for systematic reviews of preclinical animal interventions studies; 2016. Available from: www.radboudumc.nl/getmedia/4b1cbcb8-d9b6-45d5-b9fe-c92e43ab1dd4/SYRCLE-starting-guide-tool.aspx.

[25] Moher D, Liberati A, Tetzlaff J, Altman DG, Grup P (2010). Preferred reporting items for systematic reviews and meta-analyses: the PRISMA statement. Int J Surg.

[26] Sterne JAC, Savovic J, Page MJ, Elbers RG, Blencowe NS, Boutron I (2019). RoB 2: a revised tool for assessing risk of bias in randomised trials. BMJ.

[27] Wells GA, Shea B, O’Connell D, Peterson J, Welch V, Losos M, Tugwell P. The Newcastle-Ottawa Scale (NOS) for assessing the quality of nonrandomised studies in meta-analyses. 2013. Available from: http://www.ohri.ca/programs/clinical_epidemiology/oxford.asp.

[28] Hooijmans CR, Rovers MM, de Vries RB, Leenaars M, Ritskes-Hoitinga M, Langendam MW (2014). SYRCLE's risk of bias tool for animal studies. BMC Med Res Methodol.

[29] Hou C, Wu X, Jin X (2008). Autologous bone marrow stromal cells transplantation for the treatment of secondary arm lymphedema: a prospective controlled study in patients with breast cancer related lymphedema. Jpn J Clin Oncol.

[30] Maldonado GEM, Perez CAA, Covarrubias EEA, Cabriales SAM, Leyva LA, Perez JCJ (2011). Autologous stem cells for the treatment of post-mastectomy lymphedema: A pilot study. Cytotherapy.

[31] Toyserkani NM, Jensen CH, Sheikh SP, Sorensen JA (2016). Cell-assisted lipotransfer using autologous adipose-derived stromal cells for alleviation of breast cancer-related lymphedema. Stem Cells Transl Med.

[32] Toyserkani NM, Jensen CH, Andersen DC, Sheikh SP, Sorensen JA (2017). Treatment of breast cancer-related lymphedema with adipose-derived regenerative cells and fat grafts: a feasibility and safety study. Stem Cells Transl Med.

[33] Ismail AM, Abdou SM, Abdelnaby AY, Hamdy MA, El Saka AA, Gawaly A (2018). Stem cell therapy using bone marrow-derived mononuclear cells in treatment of lower limb lymphedema: a randomized controlled clinical trial. Lymphat Res Biol.

[34] Toyserkani NM, Jensen CH, Tabatabaeifar S, Jorgensen MG, Hvidsten S, Simonsen JA (2019). Adipose-derived regenerative cells and fat grafting for treating breast cancer-related lymphedema: lymphoscintigraphic evaluation with 1 year of follow-up. J Plast Reconstr Aesthetic Surg..

[35] Conrad C, Niess H, Huss R, Huber S, von Luettichau I, Nelson PJ (2009). Multipotent mesenchymal stem cells acquire a lymphendothelial phenotype and enhance lymphatic regeneration in vivo. Circulation.

[36] Hwang JH, Kim IG, Piao S, Lee DS, Lee TS, Ra JC (2011). Therapeutic lymphangiogenesis using stem cell and VEGF-C hydrogel. Biomaterials.

[37] Zhou H, Wang M, Hou C, Jin X, Wu X (2011). Exogenous VEGF-C augments the efficacy of therapeutic lymphangiogenesis induced by allogenic bone marrow stromal cells in a rabbit model of limb secondary lymphedema. Jpn J Clin Oncol.

[38] Shimizu Y, Shibata R, Shintani S, Ishii M, Murohara T (2012). Therapeutic lymphangiogenesis with implantation of adipose-derived regenerative cells. J Am Heart Assoc.

[39] Park HS, Jung IM, Choi GH, Hahn S, Yoo YS, Lee T. Modification of a rodent hindlimb model of secondary lymphedema: Surgical radicality versus radiotherapeutic ablation. BioMed research international. 2013;2013 (no pagination)(208912). 10.1155/2013/208912 PMC3856125 24350251

[40] Kawai Y, Shiomi H, Abe H, Naka S, Kurumi Y, Tani T (2014). Cell transplantation therapy for a rat model of secondary lymphedema. J Surg Res.

[41] Ackermann M, Wettstein R, Senaldi C, Kalbermatten DF, Konerding MA, Raffoul W (2015). Impact of platelet rich plasma and adipose stem cells on lymphangiogenesis in a murine tail lymphedema model. Microvasc Res.

[42] Yoshida S, Hamuy R, Hamada Y, Yoshimoto H, Hirano A, Akita S (2015). Adipose-derived stem cell transplantation for therapeutic lymphangiogenesis in a mouse secondary lymphedema model. Regen Med.

[43] Gousopoulos E, Proulx ST, Bachmann SB, Scholl J, Dionyssiou D, Demiri E (2016). Regulatory T cell transfer ameliorates lymphedema and promotes lymphatic vessel function. JCI insight.

[44] Hayashida K, Yoshida S, Yoshimoto H, Fujioka M, Saijo H, Migita K (2017). Adipose-derived stem cells and vascularized lymph node transfers successfully treat mouse hindlimb secondary lymphedema by early reconnection of the lymphatic system and lymphangiogenesis. Plast Reconstr Surg.

[45] Beerens M, Aranguren XL, Hendrickx B, Dheedene W, Dresselaers T, Himmelreich U (2018). Multipotent adult progenitor cells support lymphatic regeneration at multiple anatomical levels during wound healing and lymphedema. Sci Rep.

[46] Bucan A, Dhumale P, Jorgensen MG, Dalaei F, Wiinholt A, Hansen CR (2020). Comparison between stromal vascular fraction and adipose derived stem cells in a mouse lymphedema model. J Plast Surg Hand Surg.

[47] Dai T, Jiang Z, Cui C, Sun Y, Lu B, Li H (2020). The roles of podoplanin-positive/podoplanin-negative cells from adipose-derived stem cells in lymphatic regeneration. Plast Reconstr Surg.

[48] Ogino R, Hayashida K, Yamakawa S, Morita E (2020). Adipose-derived stem cells promote intussusceptive lymphangiogenesis by restricting dermal fibrosis in irradiated tissue of mice. Int J Mol Sci.

[49] Chen CE, Chiang NJ, Perng CK, Ma H, Lin CH (2020). Review of preclinical and clinical studies of using cell-based therapy for secondary lymphedema. J Surg Oncol.

[50] Li ZJ, Yang E, Li YZ, Liang ZY, Huang JZ, Yu NZ (2020). Application and prospect of adipose stem cell transplantation in treating lymphedema. World J Stem Cells.

[51] Vesterinen HM, Sena ES, Egan KJ, Hirst TC, Churolov L, Currie GL (2014). Meta-analysis of data from animal studies: a practical guide. J Neurosci Methods.

[52] Hadrian R, Palmes D (2017). Animal models of secondary lymphedema: new approaches in the search for therapeutic options. Lymphat Res Biol.

[53] Frueh FS, Gousopoulos E, Rezaeian F, Menger MD, Lindenblatt N, Giovanoli P (2016). Animal models in surgical lymphedema research–a systematic review. J Surg Res.

[54] Lee YJ (2020). Cell fate determination of lymphatic endothelial cells. Int J Mol Sci.

[55] Zhou C, Su W, Han H, Li N, Ma G, Cui L (2020). Mouse tail models of secondary lymphedema: fibrosis gradually worsens and is irreversible. Int J Clin Exp Pathol.

[56] Kataru RP, Baik JE, Park HJ, Wiser I, Rehal S, Shin JY (2019). Regulation of immune function by the lymphatic system in lymphedema. Front Immunol.

[57] Yuan Y, Arcucci V, Levy SM, Achen MG (2019). Modulation of immunity by lymphatic dysfunction in lymphedema. Front Immunol.

[58] Jorgensen MG, Toyserkani NM, Jensen CH, Andersen DC, Sheikh SP, Sorensen JA (2021). Adipose-derived regenerative cells and lipotransfer in alleviating breast cancer-related lymphedema: An open-label phase I trial with 4 years of follow-up. Stem Cells Transl Med.

[59] Religa P, Cao R, Bjorndahl M, Zhou Z, Zhu Z, Cao Y (2005). Presence of bone marrow-derived circulating progenitor endothelial cells in the newly formed lymphatic vessels. Blood.

[60] Percie du Sert N, Hurst V, Ahluwalia A, Alam S, Avey MT, Baker M (2020). The ARRIVE guidelines 2.0: updated guidelines for reporting animal research. BMJ Open Sci.

